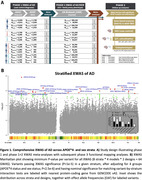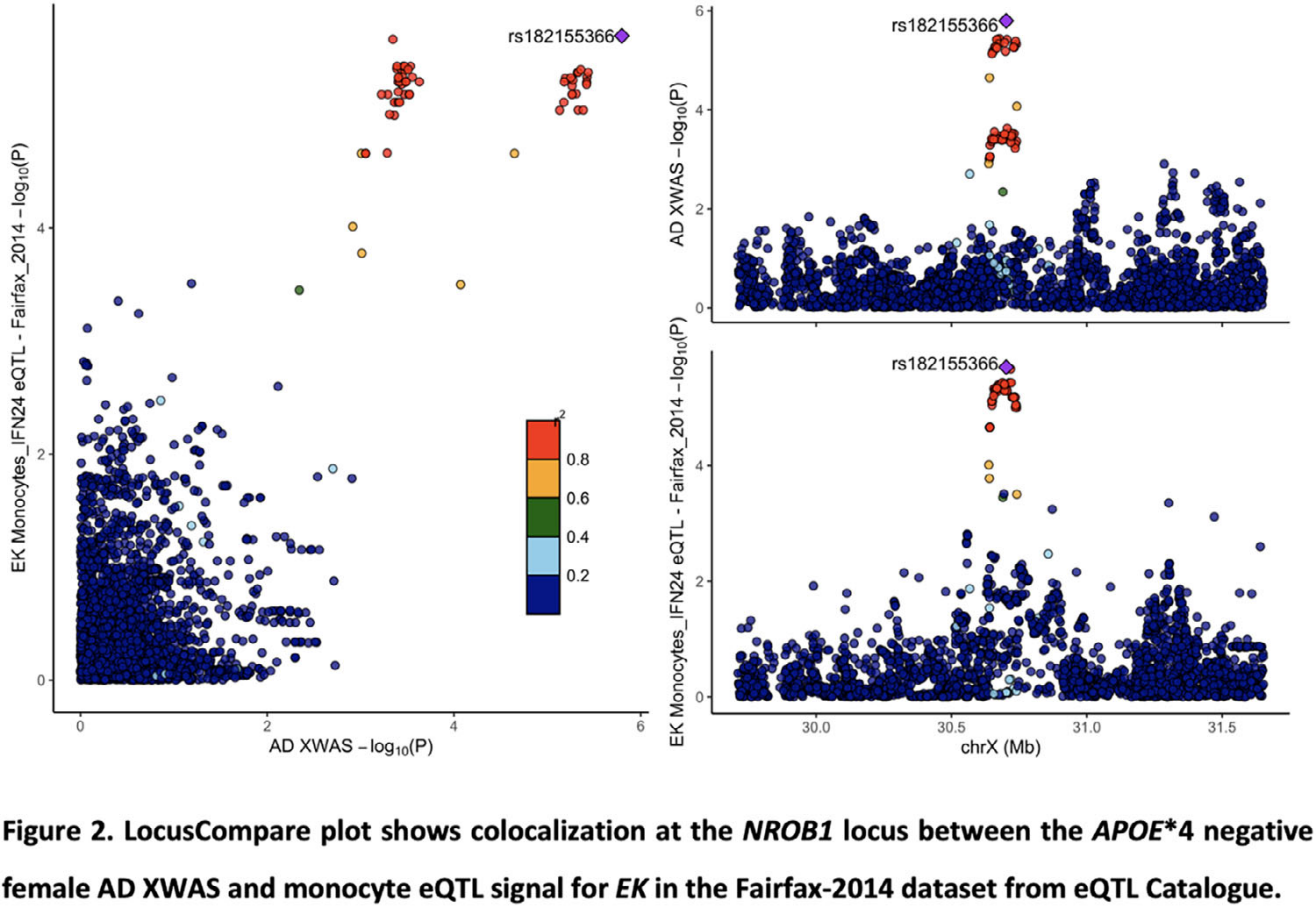# The X‐chromosome in Alzheimer’s disease genetics, stratified by APOE*4 and sex

**DOI:** 10.1002/alz.089714

**Published:** 2025-01-03

**Authors:** Michael E. Belloy, Yann Le Guen, Valerio Napolioni, Michael D Greicius

**Affiliations:** ^1^ Washington University in Saint Louis, Saint Louis, MO USA; ^2^ Stanford University, School of Medicine, Stanford, CA USA; ^3^ University of Camerino, Camerino Italy; ^4^ Stanford University, Stanford, CA USA

## Abstract

**Background:**

The X‐chromosome remains largely unexplored in Alzheimer’s disease (AD). We performed the first, stratified X‐wide association study (XWAS) of AD to chart the role of X‐chromosome genetic variation in AD sexual dimorphism and heterogeneity of APOE*4‐related AD risk.

**Method:**

The study overview is shown in **Figure 1A**. SNP‐array AD XWAS datasets primarily composing the ADGC, together with whole‐genome sequencing (WGS) from ADSP (NG00067.v7), provided case‐control diagnoses for phase‐1. The UK Biobank provided subjects with ICD codes and family history of AD status for phase‐2. Subjects were of European ancestry. Linear mixed model regressions were performed (LMM‐BOLT v.2.3.4) on AD outcome measures, adjusting for sex, APOE*4/APOE*2 dosage, genetic principal components, and array/batch/center. XWAS hits (**cf. Figure 1**) were evaluated for interaction effects (meta‐regression for phase1+2 meta‐analyses) and assessed in functional follow‐up analyses, including colocalization with QTL datasets from the eQTL Catalogue.

**Result:**

Stratified AD XWAS identified 17 loci (**Figure 1B**). These included *KDM6A*, which has previously been shown as an important risk gene for AD through rodent work. The *MAP7D3* locus contains *NHE6*, which has also been implicated as a possibly important AD risk gene. The variant rs6629203, with a rare allele frequency of 0.3%, in the *REPS2* locus reached genome‐wide significance (P<5e‐8) in APOE*4 negative individuals. At the *NROB1* locus, the AD XWAS signal and eQTL signal on the EK gene, as expressed in monocytes, colocalized (PP4 = 0.77) (**Figure 2**). Other functional follow‐up results will be shared during the conference.

**Conclusion:**

For the first time, we identified X‐chromosome loci/variants differentially associated with AD risk across APOE*4 and/or sex. Our findings contribute to our understanding of the genetic etiology of AD, which in turn contributes to advancing personalized genetic medicine. Additional follow‐up and replication work is in progress.